# The Effectiveness of *Rhodiola rosea* L. Preparations in Alleviating Various Aspects of Life-Stress Symptoms and Stress-Induced Conditions—Encouraging Clinical Evidence

**DOI:** 10.3390/molecules27123902

**Published:** 2022-06-17

**Authors:** Emilija Ivanova Stojcheva, José Carlos Quintela

**Affiliations:** Natac Biotech S.L., 28923 Madrid, Spain; jcquintela@natacgroup.com

**Keywords:** *Rhodiola rosea*, roseroot, golden root, medicinal plants, phytotherapy, clinical studies, adaptogen, stress protection

## Abstract

*Rhodiola rosea* L. has a long history of use in traditional medicine to stimulate the nervous system, treat stress-induced fatigue and depression, enhance physical performance and work productivity and treat gastrointestinal ailments and impotence. Apart from its well-established traditional use, a significant number of publications on the clinical efficacy of various *R. rosea* preparations can be found in the literature. The majority of these studies are related to the efficacy of *R. rosea* in terms of cognitive functions and mental performance, including various symptoms of life-stress, fatigue and burnout. The beneficial effects of this medicinal plant on enhancing physical performance have also been evaluated in professional athletes and non-trained individuals. Moreover, even though most evidence originates from pre-clinical trials, several clinical studies have additionally demonstrated the remediating effects of *R. rosea* on cardiovascular and reproductive health by addressing non-specific stress damage and reversing or healing the disrupted physiologies and disfunctions. Overall, in accordance with its aim, the results presented in this review provide an encouraging basis for the clinical efficacy of *R. rosea* preparations in managing various aspects of stress-induced conditions.

## 1. Introduction

*Rhodiola rosea* L. (syn. *Sedum rhodiola* DC.; *Sedum roseum* (L.) Scop), also known as “roseroot”, “golden root” or “arctic root”, belongs to the plant family Crassulaceae. The yellow-flowered herbaceous perennial naturally grows at high altitudes in dry sandy soil, on sea cliffs and in the crevices of mountain rocks of the Arctic regions of Europe and Asia (mainly Siberia), as well as the eastern coastal regions of North America [1]. *R. rosea* L. has appeared as a valuable medicinal plant in the traditional and popular medicine of a number of European and Asian countries, including Sweden, Norway, France, Germany and Iceland, as well as Russia and China. Traditionally, *R. rosea* has been used for centuries to increase physical endurance, work productivity, longevity, resistance to high-altitude sickness, to treat fatigue, depression, anemia, impotence, for gastrointestinal ailments, infections and nervous system disorders [2,3,4,5]. 

The long, well-established traditional medicinal use of *R. rosea* has stimulated extensive modern scientific research leading to the identification of *R. rosea* as an “adaptogen”, a substance that nonspecifically increases the resistance of an organism, does not disturb normal biological parameters and has a normalizing influence on physiology [6,7]. The term adaptogen dates to 1947 and has been credited to the Russian scientist Nikolai Lazarev, who defined it as an agent that allows for an organism to counteract adverse physical, chemical, or biological stressors by generating non-specific resistance [6]. To successfully combat stress and stressful situations, adaptation is required. Adaptation might be best thought of as an organism’s ability to resist a stressor by responding with either decreased or no characteristic perturbations in homeostasis. Plant adaptogens have the capacity to guide the physiological processes to start the generalized adaptation process (non-specific resistance) and handle the stressful situation in a more resourceful manner. 

Based on its long-term use in traditional medicine and numerous scientific studies, in 2011, the European Medicines Agency’s (EMA) herbal monograph on *Rhodiola rosea* L. rhizoma et radix (EMA/HMPC/232091/2011) approved its traditional use as an adaptogen for the temporary relief of symptoms associated with stress, such as fatigue, exhaustion and a general sensation of weakness [7]. The final report of the agency concluded that the long-standing use, as well as the outcome of the clinical trials, supported the plausibility of the use of *R.* *rosea*’s herbal preparation in the proposed indication. At present, scientific research and clinical studies, which are predominantly conducted in Russia, Scandinavia, Germany, UK, China, USA and other countries, have largely confirmed that *R. rosea* is an effective psychostimulant, general strengthener and an anti-stress agent. This was successfully referenced in studies addressing stress-related depression and anxiety, fatigue, cardiovascular disease, physical strength and endurance, impotence, high-altitude sickness and nervous system disorders. As a dietary supplement, numerous preparations of *R. rosea* are used worldwide. The functional claim of *R. rosea* dietary supplements that are currently mentioned in the consolidated list of Article 13 health claims of the European Food Safety Authority (EFSA) is formulated as follows: “contributes to optimal mental and cognitive activity” [8]. 

## 2. Phytochemical Composition of *R. rosea* L.

Investigations of the chemical composition of *R. rosea* rhizomes revealed at least 140 isolated compounds [2,9], including the presence of six distinct groups of phytochemicals (Table 1).

Between them, salidroside (rhodioloside), the trans-cinnamyl alcohol glycoside compounds (rhodiolin, rosin, rosavin, rosarin, and rosiridin), and tyrosol are thought to be the most critical plant constituents needed for therapeutic activity [1,2]. A characteristic of *R. rosea* is its unique presence and the relatively high rosavins content, which have not been detected in other *Rhodiola* species, and are now an accepted marker for genetically pure *R. rosea* (and its extracts) [6]. The extracts of the rhizomes of *R. rosea* are generally standardized to a minimum of 3.0% rosavins and 1.0% salidroside (in their naturally occurring ratio of 3:1), which serve as analytical markers. However, *R. rosea* rhizome extracts include an entire spectrum of chemical constituents, and the precise identification of the phytocompounds responsible for the numerous health benefits of *R. rosea* remains to be confirmed.

## 3. Pharmacological Effects and Clinical Studies

### 3.1. Neuroprotective Effects 

#### 3.1.1. Asthenia and Resilience 

The clearest indication for *R. rosea* extracts’ administration is the management of asthenic conditions, including: a decline in work performance due to physical or intellectual strain, mental and physical fatigue, sleep disturbances, poor appetite, irritability, hypertension, headaches, etc. [1,2,4]. The extract from the rhizomes of *R. rosea* acts as an adaptogen that aims to increase the body’s resistance to the imposed stressors and has a normalizing effect independent of the nature, either environmental or emotional, of the stress signal [6]. The plant’s dual actions of cognitive stimulation and emotional calming create benefits for both immediate cognitive and memory performance, as well as for the long-term preservation of brain functions. This is the main adaptogen given the indication ‘stress’ by the EMA [7]. A number of clinical trials have supported these effects.

Darbinyan et al. [10] investigated the effect of the chronic administration of 170 mg of standardized *R. rosea* rhizome extract on aspects of mental performance and fatigue on 56 healthy male and female physicians (age 24–35) on night duty for 14 days. In a randomized, placebo-controlled, double-blind, cross-over study with a wash-out period, total mental performance was measured by calculating a Fatigue Index that reflected on the outcomes of complex perceptive and cognitive cerebral functions, such as associative thinking, attention capacity, speed of visual and auditory perception and short-term memory. A statistically significant improvement in the Fatigue Index was observed in the *R. rosea* treatment group. Additionally, the improved mental performance reverted to baseline values during the washout period, supporting the beneficial effects of the extract. Among people suffering from life-stress symptoms, *R. rosea* treatment over four weeks resulted in an overall therapeutic effect with clinically relevant improvements in stress symptoms, stress-related disabilities in work, social and family life and functional impairment. In this single-arm, multicenter study by Edwards et al. [11], various aspects of stress symptoms and psychological well-being were evaluated in 101 adult subjects with life-stress symptoms who received *R. rosea* root extract (200 mg, twice-daily). All outcome measurements showed significant, consistent and steady improvements in stress symptoms, fatigue, quality of life, mood, concentration, disability, functional impairment and an overall therapeutic effect. 

In two separate studies, Spasov and colleagues investigated the effects of *R. rosea* on students during their final exam period [12,13]. During the first study, 40 medical students were randomized to receive either 50 mg of a standardized *R. rosea* extract or placebo twice daily for a period of 20 days [12]. The students receiving *R. rosea* demonstrated significant improvements in physical fitness, neuro-motoric functions, mental performance, and general well-being. Statistically significant reductions in mental fatigue, including improved sleep patterns and a reduced need for sleep, greater mood stability and a greater motivation to study were also reported in the treatment group. In a follow-up, double-blind, placebo-controlled study by the same group, 60 students received either 660 mg/day *R. rosea* extract, placebo, or nothing for 20 days [13]. The administration of *R. rosea* resulted in an increase in the physical work capacity, coordination, kinesthetic sensitivity, and general well-being of the students, along with a decrease in fatigue and situational anxiety. 

A randomized, double-blind, placebo-controlled, parallel-group clinical study by Shevtsov et al. compared the effect of a single dose of either 370 mg or 555 mg of standardized *R. rosea* extract to placebo on the capacity for mental work against a background of fatigue and stress in 161 cadets aged from 19 to 21 years [14]. The study showed a pronounced anti-fatigue effect in both *R. rosea* treatment groups, with no significant difference between the two dosages. Additionally, the levels of stress and fatigue were measured by Schutgens et al. in a double-blind, placebo-controlled clinical trial [15]. Thirty subjects were randomly assigned to three groups: a placebo group, a group that took 144 mg of *R. rosea* extract, and a group that took a supplement called ADAPT-232, a fixed combination of *Eleutherococcus senticosus* Rupr. and Maxim., *R. rosea* L. and *Schisandra chinensis* (Turcz.) Baill. After 1 week of supplementation, a significant decrease in the experienced level of fatigue was observed in the *Rhodiola* group compared with the placebo and the ADAPT-232 groups. 

*R. rosea* was shown to improve all dimensions of chronic fatigue and burnout-related symptoms. Investigating these effects of *R. rosea*, a non-interventional study was conducted in 128 primary care practices in Germany, including 330 patients with burnout indicator symptoms (exhaustion, depression, insomnia, fatigue or drop in performance). A considerable alleviation of these complaints after the administration of *R. rosea* over 8 weeks was reported, along with very good tolerability [16]. Likewise, in a double-blind, placebo-controlled clinical trial, 60 participants, selected according to the diagnostic criteria for fatigue syndrome, were randomized to receive *R. rosea* (576 mg extract/day) or placebo for four weeks [17]. *R. rosea* was found to exert a notable anti-fatigue effect that increased mental performance, particularly the ability to concentrate, and decreased cortisol response to awakening stress in burnout patients. In addition, another exploratory single-arm, multi-center study investigated the clinical outcomes of *R. rosea* intervention (200 mg of *R. rosea* extract) in 118 burnout patients [18]. The fatigue symptoms experienced by the patients continuously declined during the 8 weeks of intervention, with noteworthy results after only one week of treatment, and a statistically significant improvement at week 8. Finally, an open-label, multicenter, single-arm trial by Kasper et al. [19], explored the clinical outcomes in burnout patients treated with 400 mg of *R. rosea* extract over 12 weeks. A wide range of outcome measures assessed in the trial, such as alertness, calmness and good mood, clearly improved over time, with considerable changes already being detected after the first week of *R. rosea* administration.

The ability of *R. rosea* to increase the non-specific resistance and exert neuroprotective properties have been primarily attributed to its capacity to influence the levels and activity of several components of the stress-response system, including monoamine neurotransmitters such as serotonin and catecholamine, and opioid peptides such as β-endorphins [1,5,20,21,22,23,24]. In small and medium doses, *R. rosea* administration was found to stimulate the noradrenalin, serotonin, dopamine and acetylcholine receptors in the central nervous system (CNS) [24]. It also enhanced the effects of these neurotransmitters on the brain by increasing the permeability of the blood–brain barrier to precursors of dopamine and serotonin [25]. Additionally, the literature data indicate that *R. rosea* may stimulate the synthesis, transport, and receptor activity of opioid receptors and peptides such as the β-endorphins [23,26]. β-endorphins attenuate the intensity of the stress response and the sudden release of opioid peptides that occurs as part of the pituitary–adrenal axis response to stress. The excess production of endorphins at stressful situations interferes with normal brain functions and can lead to heart damage. In addition, *R. rosea* might protect the brain and heart by reducing the secretion of corticotrophin releasing factor (CRF) under stress, decrease corticosterone levels and increase the expression of stress-responsive genes, especially in the hippocampus and prefrontal cortex [27,28]. 

#### 3.1.2. Anxiety and Depression

Encouraging results exist regarding the use of *R. rosea* herbal preparations in treating mild-to-moderate depression, and generalized anxiety. As previously mentioned, *R. rosea* has been used in traditional medicine to alleviate the symptoms of everyday stressors, including anxiety, stress, fatigue and depression [1,2,5]. By hindering physiological stress responsivity, *R. rosea* can have a moderating effect on anxiety and mood. A pilot study by Bystritsky et al. [29] evaluated whether the extract of *R. rosea* was effective in reducing symptoms of generalized anxiety disorder (GAD). Ten participants (age 34–55 years) diagnosed with a DSM-IV of GAD were enrolled in the study, receiving a total daily dose of 340 mg *R. rosea* extract for 10 weeks. The results showed that individuals treated with *R. rosea* had a significant improvement in GAD-related symptoms, with a similar reduction in the Hamilton Anxiety Rating Scale (HAMD) scores to those found in psychopharmacological trials. Similarly, the open-label, randomized trial of Cropley et al. [30] demonstrated that participants suffering from mild anxiety responded positively to *R. rosea*. Stress and anxiety significantly decreased in the *R. rosea* treatment group during the 14-day intervention. Measures of total negative mood, anger, confusion and depression were also significantly decreased in the *R. rosea* group. The results indicated that people suffering from mild anxiety significantly benefited from *R. rosea*, while the herb also showed a protective effect against depression. 

In continuation of this, Darbinyan et al. carried out a randomized, double-blind, placebo-controlled phase III clinical trial over 6 weeks with the aim of assessing the efficacy and safety of a standardized *R. rosea* extract in patients suffering from a current episode of mild/moderate depression [31]. A total of 91 patients aged 18–70 years, with mild to moderate depression, were randomized into three groups. One received 2 *R. rosea* extract (340 mg/day) tablets each day, a second received 2 tablets twice per day, with a different dose of *R. rosea* extract (680 mg/day), and a third received 2 placebo tablets daily. For individuals in the *R. rosea* treatment groups, overall depression, together with insomnia, emotional instability and somatization, significantly improved following medication. Moreover, in both *R. rosea* treatment groups, the HAMD scores significantly improved, showing that the standardized extract from *R. rosea* possesses a clear and significant anti-depressive activity in patients suffering from mild to moderate depression. A phase II clinical trial by Mao et al. compared the efficacy of *R. rosea* extract versus sertraline, a conventional antidepressant, in treating mild-to-moderate major depressive disorder (MDD) [32]. The study included 57 participants, randomized to 12 weeks of standardized *R. rosea* extract intervention, sertraline, or placebo. Clinically meaningful odds ratios of global improvement by week 12 indicated that patients taking *R. rosea* had 1.4 times the odds of improvement, and patients on sertraline had 1.9 times the odds of improvement versus those taking placebo, indicating that *R. rosea* may possess modest antidepressant effects in patients with mild-to-moderate MDD. Although *R. rosea* showed a lower antidepressant effect compared to sertraline, it also produced significantly fewer adverse events and was much better tolerated, suggesting that *R. rosea* may possess a more favorable risk-to-benefit ratio for these individuals. Furthermore, a recent study by Gao et al. evaluated the effects of *R. rosea* in combination with sertraline in 100 patients with mild to moderate MDD for 12 weeks [33]. The results of the study showed that *R. rosea* possesses a clear and significant anti-depressive activity in patients suffering from MDD and improved their quality of life and clinical symptoms. Statistically significant reductions in the level of depression, as well as in specific symptoms of depression, such as insomnia, emotional instability and somatization, could be demonstrated. In higher doses (0.6 g/day over a 12-week period), an additional positive effect was observed. 

The molecular mechanisms involved in the antidepressant effects of *R. rosea* extracts have been examined by numerous preclinical studies [20,24,25,34,35]. A comprehensive review by Amsterdam and Panossian [25] reported that *R. rosea* stimulates the expression and release of neuropeptide-Y in neuroglial cells, controls more than 50 genes involved in the regulation of behavior, mood and depressive disorders, and is associated with certain key mediators of the stress response: regulation of the homeostasis of the hypothalamic–pituitary–adrenal (HPA) axis and modulation of G-protein-coupled receptor signaling pathways [32,34,35,36]. Moreover, other research has shown that *R. rosea* acts as an MAO-A and MAO-B inhibitor, with supporting evidence of the herb’s antidepressant and cognitive-enhancing properties [23]. In depressive rats, *R. rosea* extract could improve serotonin levels and induce neural stem cell proliferation in the hippocampus to return to normal levels, thereby repairing the hippocampus’ injured neurons [36].

### 3.2. Effects on Physical Strength and Endurance 

*R. rosea* L. has a long history of use for improving physical work capacity and physical stress endurance. Professional athletes have been effectively using *R. rosea* for several decades as a safe non-steroidal food supplement to enhance endurance and assist in rapid muscle recovery [37]. A number of scientific studies including non-athlete individuals exposed to maximal physical work, as well as professional athletes, have shown that *R. rosea* increased physical work capacity and dramatically shortened the recovery time between bouts of high-intensity exercise [38]. Animal studies suggest the mechanisms that may be involved in these effects. In mice made to swim to their limit, *R. rosea* activated the synthesis or resynthesis of ATP in mitochondria and stimulated reparative energy processes after intense exercise [39]. Moreover, chronic *R. rosea* supplementation significantly improved exhaustive swimming-induced fatigue by increasing glycogen content, the energy supply of lipogenic enzyme expressions and protective defense mechanisms [40]. In obese mice fed a high-fat diet, *Rhodiola* supplementation prevented muscle atrophy and dysfunction and activated the Sirtuin1 pathway, while atrogenes were suppressed and mitochondrial quality control was improved [41]. Finally, other studies have shown that *R. rosea* enhances cell regeneration and energy metabolism by increasing the synthesis of adenosine triphosphate, ribonucleic acid, protein, and amino acids [42]. 

Considering human trials, in a recent study by Williams et al., the effects of short-term golden root extract (*R. rosea* L.) supplementation on blood lactate, catecholamines and performance during repeated bench-press exercises were examined [43]. In a double-blinded, crossover, counterbalanced study design, 10 resistance-trained males were supplemented with either 1500 mg/day of *R. rosea* extract or placebo for 3 days. An additional 500 mg dose was ingested 30 min prior to testing. The findings indicated that short-term *R. rosea* supplementation increased the mean bench press velocity; thus, supplementation with *R. rosea* may enhance explosive resistance training performance. In another study examining the effects of an acute oral dose of *R. rosea* on endurance exercise performance, aspects of perceived exertion (RPE), mood, and cognitive function were evaluated [44]. Subjects (n = 18) ingested either *R. rosea* extract or a carbohydrate placebo 1 hour before testing in a double-blind, random crossover manner. Exercise testing consisted of a standardized 10-minute warm-up, followed by a 6-mile time trial on a bicycle ergometer. Perceived exertion, blood lactate concentration, salivary cortisol, and salivary α-amylase were measured, and a Profile of Mood States questionnaire and a Stroop Color Test were completed before warm-up and after the trial. The results of the study showed that acute *R. rosea* ingestion decreases the heart-rate response to submaximal exercise and improves endurance exercise performance by decreasing the perception of effort. The higher concentration of salivary α-amylase in the *R. rosea* treatment group suggests increased activation of the sympathetic nervous system’s response to stress. The results indicate obvious beneficial implications for recreational athletes who may choose to compete in endurance events. The acute *R. rosea* ingestion on substrate utilization, mood state, RPE, and exercise affect were also examined by Duncan et al. [45]. Ten young males completed two 30-minute cycling trials following the ingestion of either 3mg⋅kg^−1^ body mass of *R. rosea* or placebo using a double-blind, crossover design. The results revealed that the ingestion of *R. rosea* favorably influenced RPE and exercise affect without changes in energy expenditure or substrate utilization during submaximal cycling performance in regularly active adults. These changes support the efficacy of acute *R. rosea* ingestion in positively enhancing psychophysiological responses to submaximal exercise performance. 

The effects of chronic supplementation of *R. rosea* on mental and physical performance, as well as hormonal and oxidative stress biomarkers, were investigated by Jowko et al. [46] in a double-blind, randomized trial. A total of 26 (13/13) male, healthy, physical-education students received either 600 mg of *R. rosea* extract or placebo for 4 weeks, underwent psychomotor tests, and performed two incremental cycle ergometer tests of volitional fatigue. Blood samples were drawn before and after the test to measure the hormonal profile (cortisol, testosterone, and growth hormone) and biomarkers of oxidative stress (lipid hydroperoxides, total antioxidant capacity, and superoxide dismutase) and muscle damage (creatine kinase). The results indicated that chronic *R. rosea* ingestion improved psychomotor performance (simple and choice reaction time) in young, healthy, and physically active men, but did not enhance physical performance. No changes were observed in the hormonal profile; however, *R. rosea* ingestion raised the plasma’s total antioxidant capacity.

Considering anaerobic exercise performance, a study by Ballmann et al. investigated the effects of short-term *R. rosea* extract supplementation on repeated Wingate performance [47]. Eleven physically active college-aged females were supplemented with either 1500 mg/day of *R. rosea* extract or placebo for 3 days, and an additional 500 mg dose 30 min prior to testing. During each exercise trial, participants completed 3 × 15-s Wingate Anaerobic Tests (WAnTs). Over the three WAnTs, the outcome parameters of mean watts, mean anaerobic capacity, mean anaerobic power, mean peak watts, and mean total work were all higher in the *R. rosea* treatment group versus placebo. The results indicated that *R. rosea* supplementation enhanced anaerobic exercise performance by improving power output and total work during repeated WAnTs, and that this plant may possess ergogenic benefits. 

Finally, a clinical trial performed by De Bock and colleagues examined the acute and long-term effects, respectively, of *R. rosea* on exercise performance [48]. In this study, endurance capacity was the primary outcome, and muscle strength, speed of limb movement, reaction time and sustained attention were secondary outcomes. In the first study, on acute effects, *R. rosea* or placebo were taken on each of the 2 days, while the long-term effects evaluated the same outcomes over a four-week period. The results showed that three out of the six parameters of endurance capacity (time to exhaustion, O_2_ uptake and CO_2_ output) significantly improved in the *R. rosea* group. The authors concluded that acute *R. rosea* intake can improve the endurance exercise capacity in young, healthy volunteers. The most important clinical trials reporting on the effectiveness of *R. rosea* in increasing mental and physical performance are presented in Table 2. 

### 3.3. Cardioprotective Effects 

The cardioprotective effects of *R. rosea* have been widely investigated in pre-clinical studies, and between others, include the following: prevention of stress-induced cardiac damage, decreased levels of myocardial catecholamines and cyclic adenosine monophosphate (cAMP), and reduced adrenal catecholamine release [1,21,22,23]. Additionally, it has been found that *R. rosea* was able to activate the mu-opiate receptors in animal heart muscle preventing reperfusion arrhythmias [49]. This effect could be blocked by naloxone injection (known to inhibit mu-opiate receptors), thus suggesting that the anti-arrhythmic effect of *R. rosea* is associated with mu-opiate receptors in myocardial muscle. Using the same mechanism, *R. rosea* extract supplementation decreased the systolic blood pressure and heart-rate in a dose-dependent manner in spontaneously hypertensive mice [23]. Furthermore, the oral administration of *R. rosea* oligomeric proanthocyanidins in atherosclerosis rats improved the progress of atherosclerosis by regulating the lipid metabolism, restoring the antioxidant capacities, attenuating pro-inflammatory cytokines and chemocytokines release, and improving the endothelial dysfunction indicated by the nitric oxide system [50]. It has been suggested that *R. rosea* offers some cardioprotective benefits that are not associated with other adaptogens. Its proposed ability to moderate stress-induced damage and dysfunction in cardiovascular tissue might make *R. rosea* the adaptogen of choice among patients at higher risk of cardiovascular disease [6]. 

In a study by Parisi et al. [51], the effects of chronic *R. rosea* supplementation on sport performance and antioxidant capacity in male athletes were examined. Following a chronic supplementation with *R. rosea* for 4 weeks, 14 trained male athletes underwent a cardio-pulmonary exhaustion test and blood samples were taken to evaluate their antioxidant status and several biochemical parameters. Prolonged *R. rosea* supplementation was able to reduce both lactate levels and parameters of skeletal muscle damage after an exhaustive exercise session in the athletes. Moreover, this intake reduced, in a statistically significative manner, plasma free fatty acids levels, indicating that *R. rosea* supplementation seems to ameliorate fatty acid consumption. Skarpanska-Stejnborn et al. investigated the effects of a 4-week intervention with *R. rosea* extract on the balance of oxidants and antioxidants in the serum and erythrocytes of competitive rowers [52]. In a double-blinded study, 22 members of the Polish Rowing Team performed a 2000-m maximum test on a rowing ergometer. Several enzymatic redox parameters were assessed in erythrocytes, creatine kinase activity and total antioxidant capacity were measured in plasma samples, lactate levels were determined in capillary blood samples, and uric acid concentrations were measured in serum. After supplementation, the total plasma antioxidant capacity was significantly higher in the supplemented group compared to the placebo group, and superoxide dismutase activity in erythrocytes directly after and 24 h after the ergometry was significantly lower in the athletes who received the *R. rosea* extract. 

A three-arm, double-blind clinical study by Abidov et al. compared the effects of *R. rosea* extract supplementation on the blood levels of inflammatory C-reactive protein (CRP) and creatinine kinase (CK) in healthy untrained volunteers before and after exhausting exercise to placebo, or nothing [37]. The study examined muscle recovery in 36 healthy untrained adults that underwent an exhausting physical exercise test on day 30, which consisted of cycling on a bicycle ergometer with gradual power increases until volitional exhaustion. The findings indicated that *R. rosea* significantly lowered the CRP levels at 5 h and 5 days after the test (*p* < 0.05), while the CK levels were much lower in the R. rosea treatment group 5 days after the test and indicated a decrease towards the initial levels. It was concluded that the use of *R. rosea* extract could facilitate recovery after exercise and decrease the risk of cardiological disorders, in line with the cardioprotective properties of this plant. Lastly, a systematic review and meta-analysis conducted by Yu et al., including 13 randomized controlled trials involving 1672 participants, evaluated the efficacy and safety of various *Rhodiola* formulations in treating ischemic heart disease [53]. Overall, the results showed that the effectiveness of *Rhodiola* formulations was higher compared to other medicines in the control groups, with statistically significant differences observed in both symptomatic improvement and electrocardiography improvement. The authors concluded that *Rhodiola* formulations may have a positive effect on treating ischemic heart disease alone and in combination with routine western medicine.

### 3.4. Reproductive Effects 

Today, the role of stress has been acknowledged as being the underlying cause for many disease processes [35]. Stress is commonly overlooked as an obstacle to natural conception, despite the fact that stress can shut down the activity of the hypothalamic–pituitary–gonadal axis, which controls the reproductive system [54]. This can disrupt the connection between the brain and the ovaries and cause delayed or absent ovulation and irregular or missed menses. Better managing stress and initiating the relaxation response can help to reverse or heal the disrupted physiology that is causing infertility [55]. Traditional medicine transcripts report that *R. rosea* has been given to newlyweds in Siberia to boost fertility. In some mountain villages of the Republic of Georgia, a bouquet of roots is still given to couples prior to marriage to enhance fertility and assure the birth of healthy children [1,5,25]. At present, clinical trials supporting these effects of *R. rosea* are still extremely scarce, but those reported have nonetheless shown that *R. rosea* extracts are able to restore ovulation in women with amenorrhea (loss of menstrual cycle) and physicians have reported cases of women who failed to conceive using fertility drugs becoming pregnant after taking *R. rosea* [1,56,57].

Several animal studies have demonstrated the effect of *R. rosea* in fertility and sexual function. *R. rosea* enhanced egg maturation in female rats and produced an anabolic effect in the males of a number of species (increased muscle-building and gonad-strengthening similar to the effects of low-dose testosterone) [1]. In the majority of *R. rosea*-treated animals, the number of growing follicles, the oocyte’s volume, the accumulation of RNA in the oocyte’s cytoplasm, the proliferation of the lining and glandular cells of the uterine horns, and the preparation of uterine mucosa for fertilization all increased. The administration of *R. rosea* extract to sexually mature female mice over a period of 4 weeks of prolonged menstruation from 1.3 days (control) to 2.8 days (*R. rosea* treated), reduced the resting period from 3.8 days (control) to 2.2 days (*R. rosea*-treated), and increased the relative number of estrous days from 29% to 56%. Additionally, *R. rosea* increased the mean weight of the uterine horns and the mean weight of the ovaries [56,57]. A recent study by Kadioglu et al. investigated the effect of *R. rosea* root extract on stress-induced ovarian damage, infertility and reproductive disorders in female rats [58]. It was found that taking *R. rosea* extract had beneficial effects on the treatment of stress-induced reproductive dysfunction and significantly prevented the increase in oxidative parameters and proinflammatory cytokine levels in ovarian tissue. 

These pre-clinical investigations led to a clinical study investigating the effects of *R. rosea* extract in women suffering from amenorrhea [5,56,57]. Forty women with amenorrhea were given *R. rosea* extract (either 100 mg of dried *R. rosea* extract orally twice a day for 14 days, or 1 mL liquid *R. rosea* extract intramuscularly for 10 days). In some participants, the treatment cycle was repeated 2–4 times. Normal menses was restored in 25 women, 11 of whom became pregnant. In those with normal menses, the mean length of the uterine cavity increased from 5.5 cm to 7.0 cm (considered normal) after *R. rosea* treatment. In line with this study, a recent four-arm parallel group, placebo-controlled, randomized, double-blind, clinical trial assessed the efficacy and safety of an herbal preparation named Menopause relief EP^®^, a hybrid combination of *Actaea racemosa* L. (black cohosh, BC) and *R. rosea* (RR) root extracts in women with menopausal complaints [59]. A total of 220 women were randomly assigned to receive either BC (6.5 mg), BC500 (500 mg), Menopause Relief EP^®^, or placebo once per day for 12 weeks. The study found that the menopause-symptom-relief effects of the BC-RR combination were significantly superior in all tests to the effects of BC alone and placebo. The combination BC-RR significantly improved the menopausal quality of life, mainly due to its superior effect on emotional and physical health domains. Finally, in an open study, 35 men with erectile dysfunction and/or premature ejaculation (of 1–20 years duration) took 150–200 mg/day of *R. rosea* extract for 3 months [60]. A total of 26 of 35 men (74%) responded to *R. rosea,* with substantially improved sexual function, normalization of prostatic fluid, and an increase in 17-ketosteroids in urine.

## 4. Conclusions

*Rhodiola rosea* L. has a well-established use in the traditional medicinal systems of the Nordic countries, Eastern Europe and Asia, with a reputation for enhancing mental and physical performance, decreasing symptoms of fatigue and depression, increasing work productivity, and providing antioxidant and anti-inflammatory effects, among others. Current research has clearly established *R. rosea* as a phytomedicinal plant with adaptogenic effects, which is able to enhance the body’s non-specific resilience to physical and mental stresses and normalize its functions. Stress has been associated with an increased incidence of numerous health disorders of the neuroendocrine-immune system, from milder physiological conditions such as a decrease in physical and mental capacity and a sensation of weakness, to more concerning health conditions such as anxiety, depression, fatigue and burnout, cardiovascular and reproductive disfunctions. The long history of medicinal use and the extensive pre-clinical and clinical research into its stimulant and stress-protective properties have undoubtably validated the effectivity of using *R. rosea* L. to address all the above-mentioned stress-induced conditions and disorders. 

## Figures and Tables

**Table 1:** Main phytochemicals identified in *Rhodiola rosea* L.

Group of Compounds	Compound
Phenylpropanoids	rosavin, rosin, rosarin collectively known as **rosavins**
Phenylethanoid derivatives	**salidroside** (rhodioloside), tyrosol
Flavanoids	rhodiolin, rhodionidin, rhodionin, rhodiosin, rhodalidin, tricin
Monoterpene derivatives	rosiridol, rosiridin, rhodiolosides A–E
Triterpenes	daucosterol, beta-sitosterol
Phenolic acids	chlorogenic acid, hydroxycinnamic acid, gallic acid

**Table 2:** Relevant clinical trials on the effects of Rhodiola rosea L on mental and physical performance.

Reference	Study Design	Condition	Participants	Intervention	Control	Duration	Outcome Measures	Results
Darbinyan (2000)	randomized, placebo-controlled, double-blind, crossover with a wash-out period	Work-related fatigue	56 healthy male and female physicians aged 24–35	170 mg of *R. rosea* SHR-5 extract	Placebo	42 days total duration; 14 days intervention	Overall level of mental fatigue calculated according to the Fatigue Index, involving complex perceptive and cognitive cerebral functions	A statistically significant improvement in the Fatigue Index observed in the *R. rosea* treatment group compared to placebo
Edwards (2012)	multicentre, non-randomized, open-label, single-arm study conducted in 13 centres in UK	Life-stress symptoms	101 ambulatory subjects between 30 and 60 years of age with life-stress symptoms	200 mg of *R. rosea* dry extract WS^®^ 1375, twice daily	None	4 weeks	(1) Numerical Analogue Scales (NAS) of subjective stress Symptoms; (2) Perceived Stress Questionnaire (PSQ); (3) Multidimensional Fatigue Inventory 20 (MFI-20); (4) Numbers Connecting Test (NTC); (5) Sheehan Disability Scale (SDS); (6) Multidimensional Mood State Questionnaire (MDMQ); and (7) Clinical Global Impressions (CGI)	All outcome variables showed consistent and steady improvements with regard to stress symptoms, fatigue, quality of life, mood, concentration, disability, functional impairment and overall therapeutic effect. A statistically significant improvement between baseline and week 4 based on the two-sided Wilcoxon signed-rank test was observed
Spasov (2000)	randomized, double-blind, placebo-controlled, 2 parallel groups study	Non-specific fatigue and stress during examination	40 healthy medical students, 17–19 years old	50 mg of *R. rosea* SHR-5 extract, twice daily	Placebo	20 days	Physical fitness measured as: physical work capacity and an increase in pulse rate, neuro-motoric fitness, mental work capacity, self-evaluation of fatigue and general well-being	The students receiving *R. rosea* demonstrated significant improvements in physical fitness, neuro-motor functions, mental performance, and general well-being
Spasov (2000)	randomized, placebo-controlled, 3 parallel groups study	Study-related fatigue and stress	60 healthy male students, 17–18 years old	660 mg of *R. rosea* extract with no ethyl alcohol	Placebo and untreated	20 days	Psychological fatigue, situational anxiety, motivation, precision of motor function, process of excitement, need for rest, mental work capacity and neuromotor function	An increase in physical work capacity, coordination, kinaesthetic sensitivity, and general well-being, along with a decrease in fatigue and situational anxiety observed in the *R. rosea* treatment group
Shevtsov (2003)	randomized, double-blind, placebo-controlled, parallel-group study with an extra non-treatment group	Work-related fatigue and stress	161 healthy cadets, 19–21 years old	(1) 370 mg of *R. rosea* SHR-5 extract; (2) 555 mg of *R. rosea* SHR-5 extract	Placebo and untreated	Single dose	Total Antifatigue Index (TAFI) calculated by two efficacy parameters: capacity for mental work, physiological parameters and safety parameters	A pronounced anti-fatigue effect reflected in the TAFI found in both *R. rosea* treatment groups. Statistically highly significant results for both doses.
Schutgens (2009)	randomized, double-blind, placebo-controlled, 3 parallel groups study	Experienced levels of stress and fatigue	30 healthy students; mean age: 21.1 years	144 mg of SHR-5 *R. rosea* extract, twice-daily	Placebo and ADAPT-232	7 days	(1) Ultra-weak photon emission; (2) Self-evaluated stress; (3) Self-evaluated fatigue.	A statistically significant decrease in the experienced level of fatigue and decreased photon emission observed in the *Rhodiola* treatment group
Goyvaerts (2012)	open-label design	Burnout and fatigue syndrome	330 female (74%) and male (26%) patients between 18 and 81 years	288 mg of *Rhodiola rosea* SHR-5 extract	None	8 weeks	Total burnout score: (1) Fatigue; (2) Exhaustion; (3) Depression; (4) Insomnia; (5) Loss of power.	The *Rhodiola* extract showed an impressive, highly significant and continuous decline in the complaints of up to 63%, together with very good tolerability.
Olsson (2009)	randomized, double-blind, placebo-controlled, parallel group study	Fatigue syndrome	60 patients diagnosed with fatigue syndrome; mean age: *Rhodiola* group—41 years, Placebo group—42.1 years	144 mg of *R. rosea* SHR-5 extract, 4 capsules per day	Placebo	28 days	(1) Fatigue (Pines’ burnout scale); (2) Depression (MADRS rating scale); (3) Quality of life (SF-36 questionnaire); (4) Saliva cortisol upon awakening; (5) Attention (CCPT II test).	A notable anti-fatigue effect that increased mental performance, particularly the ability to concentrate and decreased cortisol response to awakening stress in burnout patients.
Lekomtseva (2017)	open-label, single-arm, multicentre study conducted in 5 hospitals in Ukraine	Prolonged or chronic fatigue syndrome	100 subjects, 31 male, 69 female ones. Mean age: 37.8 ± 9.5 years	200 mg of *R. rosea* dry extract WS^®^ 1375, twice daily	None	8 weeks	(1) Multidimensional Fatigue Inventory 20 (MFI-20); (2) NAS of chronic fatigue symptoms; (3) NTC; (4) SDS; (5) Pittsburgh Sleep Quality Index (PSQI); (6) PAQ; (7) Beck Depression Inventory II (BDI-II); (8) (CGI).	A significant improvement in prolonged or chronic fatigue symptoms over 8 weeks. The values of nearly all outcome variables markedly improved over time, with a substantial alleviation of symptoms that could already be observed after the first week of treatment.
Kasper (2017)	exploratory, open-label, multicentre, single-arm trial conducted at four centres in Vienna, Austria	Burnout symptoms	118 outpatients aged 30–60 years suffering from burnout symptoms	A daily dose of 400 mg *R. rosea* extract (WS^®^ 1375, Rosalin)	None	12 weeks	(1) Maslach Burnout Inventory; (2) Burnout Screening Scales I and II; (3) SDS; (4) PSQ; (5) NCT; (6) MDMQ; (7) NAS for different stress symptoms and impairment of sexual life; (8) Patient Sexual Function Questionnaire; (9) CGI Scales.	The majority of the outcome measures showed clear improvements over time. A steady and substantial, statistically relevant alleviation of the majority of the assessed burnout symptoms was observed as early as 1 week after the start of treatment.
Bystritsky (2008)	open-label design	Generalized anxiety disorder (GAD)	10 participants with a DSM-IV diagnosis of GAD between the ages of 34 and 55	340 mg of *R. rosea* extract (Rhodax^®^)	None	10 weeks	(1) HARS; (2) Four-Dimensional Anxiety and Depression Scale; (3) CGI of Severity/Improvement Scale	Significant improvements in GAD symptoms were found with *R. rosea*, with a reduction in HARS scores similar to that found in clinical trials.
Cropley (2015)	open-label, randomized, repeated measures design	Self-reported anxiety, stress, cognition, and other mood symptoms	81 mildly anxious students randomized into one of two conditions: treatment = 40; control = 41 volunteers	2 × 200 mg of a dry extract from *R. rosea* roots daily (Vitano^®^)	Untreated	14 days	Primary outcomes: (1) Anxiety; (2) Stress. Secondary outcomes: (3) Mood; (4) Sleepiness; (5) Sleep; (6) Cognitive tests; (7) Choice reaction time; (8) Sustained Attention to Response Test (SART); (9) Symbol Digit Processing.	The experimental group demonstrated a significant reduction in self-reported anxiety, stress, anger, confusion and depression at 14 days and a significant improvements in total mood. No relevant differences in cognitive performance were observed.
Darbinyan (2007)	randomized, double-blind, placebo-controlled study with 3 parallel groups	Mild to moderate depression	89 male and female patients diagnosed with mild or moderate depression, age 18–70 years	2 groups: (1) *R. rosea* SHR-5 extract 340 mg/day, (2) *R. rosea* SHR-5 extract 680 mg/day	Placebo	6 weeks	(1) Beck Depression Inventory (BDI); (2) Hamilton Rating Scale for Depression (HAM-D) questionnaires.	Overall depression, insomnia, emotional instability and somatization significantly improved following medication. *R. rosea* shows anti-depressive potency.
Mao (2015)	randomized, double-blind, placebo-controlled study with 3 parallel groups	Mild to moderate major depressive disorder (MDD)	57 male and female patients: *R. rosea* (n = 20), sertraline (n = 19), placebo (n = 18). Mean age: 45 years	(1) 340 mg of *R. rosea* SHR-5 extract, or (2) sertraline 50 mg HCl	Placebo	12 weeks	(1) Hamilton Depression Rating (HAM-D); (2) Beck Depression Inventory (BDI); (3) Clinical Global Impression Change (CGI/C).	Patients taking *R. rosea* 1.4 times the odds of improvement, patients on sertraline 1.9 times the odds of improvement. *R. rosea* better tolerated than sertraline.
Gao (2020)	randomized, double-blind, placebo-controlled study with 3 parallel groups	Mild to moderate major depressive disorder (MDD)	100 patients (33/33/34) with a DSM IV Axis I diagnosis of MDD aged 18–50 years	(A) sertraline (B) sertraline and *Rhodiola* (0.6 g/day) daily, (C) sertraline and *Rhodiola* capsule tablet (0.3 g/day).	Placebo	12 weeks	(1) Hamilton Depression Rating (HAM-D); (2) Beck Depression Inventory (BDI); (3) Clinical Global Impression Change (CGI/C).	Statistically significant reductions in HAM-D, BDI, and CGI scores for all treatment conditions. The decline in HAM-D, BDI, and CGI scores was greater for group B versus group C and A.
Williams (2021)	placebo-controlled, double-blind, counterbalanced, crossover study	Resistance exercise performance	10 resistance-trained males	1500 mg/day standardized *R. rosea* extract for 3 days, additional 500 mg 30 min before testing	Placebo	3 days	(1) Performance during repeated bench press exercise; (2) Blood concentrations of lactate (LA), epinephrine (EPI), and norepinephrine (NE).	*R. rosea* increased mean bench press velocity. Higher LA and NE levels following exercise in the *R. rosea*-treated group, suggesting increased CNS and/or sympathetic activity.
Noreen (2013)	randomized, placebo-controlled, double-blind, crossover study	Endurance exercise performance, perceived exertion, mood and cognitive function	18 recreationally active college women (22 ± 3.3 years, 56.6 ± 6.2 kg)	3 mg·kg−1 of standardized *R. rosea* extract 1 hour before testing	Placebo	Single dose	(1) Rating of perceived exertion (RPE) (10-point Borg scale); (2) Blood lactate concentration, salivary cortisol, and salivary α-amylase; (3) Profile of Mood States (POMS) questionnaire and a Stroop Colour Test;	*R. rosea* ingestion decreased heart rate during the warm-up and the mean RPE in the trial. Subjects completed the test significantly faster with *R. rosea*. Higher concentration of salivary alpha amylase in the *R. rosea* treatment group.
Duncan (2014)	placebo-controlled, double-blind, crossover study	Exercise performance, substrate utilisation, mood State, and rating of perceived exertion	Ten males, recreational exercisers (mean age ± S.D. = 26 ± 6 years)	3 mg⋅kg−1 body mass of *R. rosea* (Indigo Herbs)	Placebo	Single dose	(1) Two 30-m cycling trials at an intensity of 70% of VO_2 max_; (2) Heart rate; (3) Rating of per ceived exertion (RPE); (4) mood state; (5) substrate utilisation	Ingestion of *R. rosea* favourably influenced RPE and exercise affect without changes in energy expenditure or substrate utilization during 30-m submaximal cycling performance
Jowko (2016)	randomized, double-blind, placebo-controlled trial	Mental and physical performance, hormonal and oxidative stress biomarkers	26 healthy, male, physical -education students (13/13)	600 mg of standardized *R. rosea* extract	Placebo	4 weeks	(1) Psychomotor tests for simple and choice reaction time—Vienna Test System; (2) VO_2peak_ test; (3) Hormonal profile (cortisol, testosterone, and growth hormone); (4) Biomarkers of oxidative stress; (5) Muscle damage (CK).	*R. rosea* ingestion improved psychomotor performance and shortened reaction time and total response time. No changes in endurance exercise capacity and hormonal profile were observed. *R. rosea* ingestion raised plasma total antioxidant capacity.
Ballman (2018)	randomized, blinded, placebo-controlled and counterbalanced study	Anaerobic exercise performance	11 physically active female participants, aged 18 to 24 years	1500 mg/day standardized *R. rosea* extract for 3 days, 500 mg 30 min before testing	Placebo	3 days	3 × 15 second Wingate Anaerobic Tests (WAnTs)—mean watts, anaerobic capacity, anaerobic power, mean peak watts, and mean total work	All WAnTs outcome measures were higher in the *R. rosea* treatment trial. *R. rosea* enhanced power output and total work during repeated WAnTs.
De Bock (2004)	randomized, placebo-controlled, double-blind, crossover study	Improvement in endurance exercise performance	24 healthy and physically active male and female students (12/12), mean age: male—21.8 years, female—20.2 years	2 capsules containing 100 mg of standardized *R. rosea* extract	Placebo	Phase I: 2 days Phase II: 4 weeks	(1) Endurance exercise capacity; (2) Muscle strength; (3) Speed of limb movement; (4) Reaction time; (5) Ability to sustain attention.	Time to exhaustion, O2 uptake and CO2 output significantly improved in the *R. rosea* group. Acute *R. rosea* intake can improve the endurance exercise capacity in young healthy volunteers.

SHR-5: dry extract of root and rhizome of *Rhodiola rosea* L., drug-extract-ratio 2.5–5:1, first extraction solvent ethanol 70%, second extraction solvent water. Abbreviations: HAM-D—Hamilton Depression Rating; HARS—Hamilton Anxiety Rating Scale; CGI—Clinical Global Impressions, TAFI—Total Antifatigue Index; SDS—Sheehan Disability Scale; PSQ—Perceived Stress Questionnaire; NTC—Number Connection Test; MDMQ—Multidimensional Mood State Questionnaire; NAS—Numerical Analogue Scales; BDI—Beck Depression Inventory; RPE—rating of perceived exertion; WAnTs—Wingate Anaerobic Tests; POMS—Profile of Mood States.

## Data Availability

Not applicable.
